# *Polygonum multiflorum* Inhibits Pulmonary Inflammation and Fibrosis in PM_2.5_-Induced Dysfunction Through the Regulation of the TLR4/TGF-β1 Signaling Pathway in Mice

**DOI:** 10.3390/ijms26115080

**Published:** 2025-05-25

**Authors:** Hye Ji Choi, Hyo Lim Lee, In Young Kim, Ho Jin Heo

**Affiliations:** Division of Applied Life Science (BK21), Institute of Agriculture and Life Science, Gyeongsang National University, Jinju 52828, Republic of Korea; hjchoi0820@gnu.ac.kr (H.J.C.); gyfla059@gnu.ac.kr (H.L.L.); inzero331@gnu.ac.kr (I.Y.K.)

**Keywords:** *Polygonum multiflorum*, PM_2.5_, 2,3,5,4′-tetrahydroxystilbene-2-O-β-D-glucoside, antioxidants, inflammation, respiratory dysfunction

## Abstract

Industrial development has improved living standards; however, mortality associated with fine particulate matter (PM_2.5_) exposure continues to rise. Despite increasing awareness of its health risks, effective strategies to mitigate PM_2.5_-induced pulmonary damage remain limited. This study examines the protective properties of an ethanolic extract from *Polygonum multiflorum* (EPM) in preventing pulmonary dysfunction induced by PM_2.5_, as well as its possible use as a dietary intervention to improve respiratory health. The physiological compounds in EPM were identified using ultra-performance liquid chromatography, and its protective effects were evaluated via in vitro assays using A549 and RPMI 2650 cells. The antioxidant system and mitochondrial function were further analyzed in the lung tissues of PM_2.5_-exposed BALB/c mice, with molecular mechanisms elucidated by Western blot analysis. The main bioactive compounds identified in EPM included 2,3,5,4′-tetrahydroxystilbene-2-O-β-D-glucoside. EPM modulated the Nrf2 signaling pathway, enhancing antioxidant defense by regulating the expression of antioxidant-related proteins. Furthermore, EPM exhibited protective effects against inflammation, apoptosis, and fibrosis through the TLR4/p-JNK and TGF-β1 signaling pathways. These findings suggest that EPM exerts protective effects against PM_2.5_-induced oxidative stress and inflammation and may be used as a functional food ingredient for respiratory health.

## 1. Introduction

Annually, nearly 7 million deaths are linked to air pollution globally, as reported by the World Health Organization [1]. Major pollutants contributing to this problem include particulate matter (PM), ozone, and various chemical pollutants, including carbon, sulfur, and nitrogen [1]. PM is composed of liquid and solid particles that are released directly into the atmosphere from diesel use, industrial activities, and agricultural dust. PM, originating from various sources, is categorized based on its aerodynamic size, and PM_2.5_ denotes particles that measure 2.5 μm or smaller [2]. Inorganic components of PM_2.5_ that enter the body through the bronchial airways contribute to reactive oxygen species (ROS) generation primarily via the Fenton reaction [3]. Additionally, PM_2.5_ activates alveolar macrophages in the lungs, which further increases ROS production and exacerbates inflammation [2,3]. Elevated ROS levels induce oxidative stress, leading to cellular DNA damage and protein modifications [4]. Moreover, elevated ROS levels activate Toll-like receptor (TLR) 4, which is a crucial component of the TLR family that is involved in inflammatory responses [5]. Upon activation, TLR4 recruits myeloid differentiation primary response 88 (MyD88) and initiates a signaling cascade through a series of kinases [6]. This cascade ultimately results in the activation of nuclear factor kappa-light-chain-enhancer of activated B cells (NF-κB) and the subsequent release of cytokines [6]. These cytokines promote tissue fibrosis and lung damage by upregulating transforming growth factor beta 1 (TGF-β1) expression, which is a key mediator with a pro-fibrotic activity [7]. Consequently, chronic inflammation induced by PM_2.5_ exposure through oxidative stress contributes significantly to pulmonary fibrosis and tissue damage [8]. To alleviate the health risks linked to PM_2.5_ exposure, it is essential to implement strategies focused on lowering PM_2.5_ emissions while minimizing individual contact with PM_2.5_ [9,10]. However, since completely avoiding PM_2.5_ is impossible, the intake of natural antioxidants capable of scavenging ROS is critical in relation to reducing oxidative stress and inflammatory responses induced by PM_2.5_.

*Polygonum multiflorum* (*P. multiflorum*) is a member of the *Polygonaceae* family and is traditionally used in East Asian medicine [11]. *P. multiflorum* possesses phenolic compounds, including anthraquinone, stilbene glycoside, and tannin, and exhibits various physiological effects [12]. A prior investigation demonstrated that extracts of *P. multiflorum* exhibited a dose-dependent reduction in symptoms associated with allergic asthma in a mouse model, including airway inflammation, mucus production, and airway hyperresponsiveness [13]. However, there is a lack of research on the potential benefits of *P. multiflorum* in alleviating pulmonary dysfunction resulting from chronic PM_2.5_ exposure. In particular, long-term exposure to PM_2.5_ can cause progressive lung tissue damage, leading to a gradual deterioration in pulmonary function, but there are currently limited effective treatments to prevent or alleviate this damage. In this context, the present study was conducted to assess the pulmonary protective properties of *P. multiflorum* in a PM_2.5_-induced mouse model, using doses informed by previously published in vivo studies [14,15]; its applicability as a functional food was also explored.

## 2. Results

### 2.1. Bioactive Constituents of EPM

**Figure 1:**
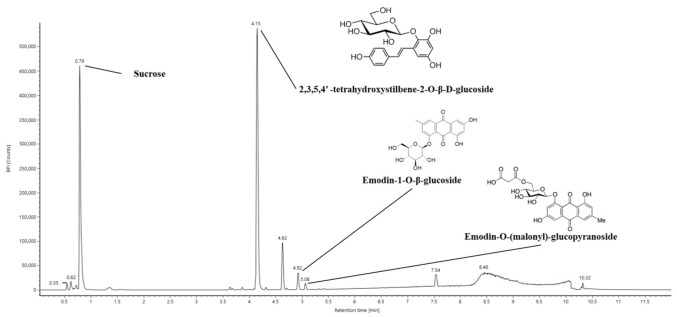
Ultra-performance liquid chromatography coupled with quadrupole time-of-flight mass spectrometry (UPLC–Q–TOF/MS^E^) chromatogram of 40% ethanolic *P. multiflorum* (EPM) extracts.

The bioactive constituents of 40% ethanolic *P. multiflorum* (EPM) were analyzed using ultra-performance liquid chromatography coupled with a quadrupole time-of-flight mass spectrometry (UPLC-Q-TOF/MS^E^) system (Figure 1). Sucrose, 2,3,5,4′-tetrahydroxystilbene-2-O-β-D-glucoside (TSG), emodin-1-O-β-glucoside, and emodin-O-(malonyl)-glucopyranoside were determined as key components of EPM (Table 1).

### 2.2. EPM Improved PM_2.5_-Induced Cytotoxicity in A549 and RPMI2650 Cells

**Figure 2:**
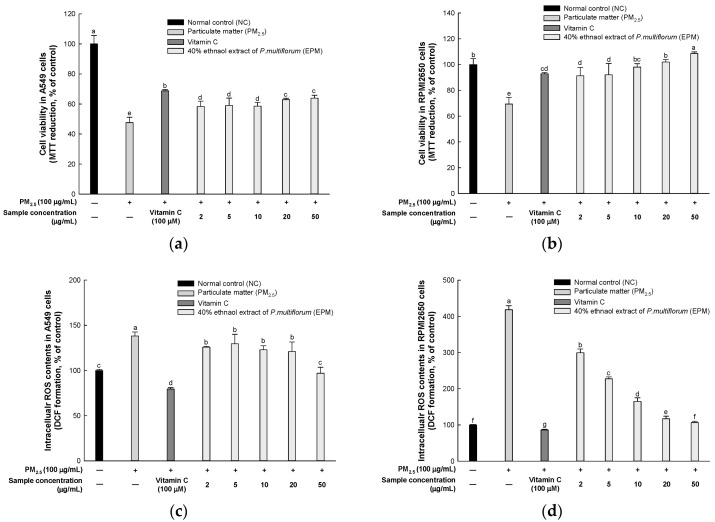
Protective effect of 40% ethanolic *P. multiflorum* (EPM) extracts in PM_2.5_-induced cytotoxicity in A549 and RPMI2650 cells. (**a**,**b**) Cell viability. (**c**,**d**) Intracellular reactive oxygen species (ROS) contents. The data were expressed as mean ± SD (*n* = 3). A one-way analysis of variance (ANOVA) test with Duncan’s multiple range tests was conducted. Distinct lowercase letters on the bar graph denote significant differences among groups (*p* < 0.05).

Cell viability was measured in A549 and RPMI2650 cells to evaluate the pulmonary protective effect of EPM (Figure 2a,b). The fine particulate matter (PM_2.5_) group exhibited a significant reduction in cell viability (47.58% and 69.39%, respectively) compared to the normal control (NC) group (100%). However, treatment with vitamin C restored cell viability to 68.73% and 92.88%, while treatment with 50 μg/mL EPM further increased it to 63.94% and 108.57%, respectively, compared to the PM_2.5_ group.

ROS contents were measured in A549 and RPMI2650 cells to evaluate the pulmonary protective effect of EPM (Figure 2c,d). The PM_2.5_ group exhibited a significantly high ROS content (138.26% and 418.39%, respectively) compared to the normal control group (100%). However, treatment with vitamin C decreased ROS contents to 79.56% and 86.27%, while treatment with 50 μg/mL EPM further enhanced it to 97.00% and 106.60%, respectively, when compared with the PM_2.5_ group.

### 2.3. EPM Alleviated the Antioxidant System in PM_2.5_-Induced Pulmonary Dysfunction

**Figure 3:**
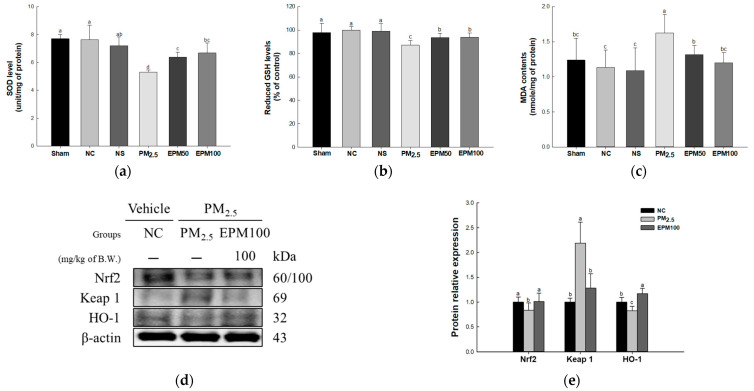
Effect of 40% ethanolic *P. multiflorum* (EPM) extracts on the antioxidant system in lung tissues of PM_2.5_-induced mice. (**a**) Superoxide dismutase (SOD) levels. (**b**) Reduced glutathione (GSH) levels. (**c**) Malondialdehyde (MDA) contents (*n* = 5). (**d**) Western blot band images. (**e**) Relative expression levels of nuclear factor erythroid-2-related factor 2 (Nrf2), Kelch-like ECH-associated protein1 (Keap1), and heme oxygenase-1 (HO-1) were quantified based on β-actin (*n* = 3). The data were expressed as mean ± SD. A one-way analysis of variance (ANOVA) test with Duncan’s multiple range tests was conducted. Distinct lowercase letters on the bar graph denote significant differences among groups (*p* < 0.05).

Superoxide dismutase (SOD) levels were reported to decrease in the PM_2.5_ group (5.30 unit/mg of protein) compared with the NC group (7.63 unit/mg of protein) (Figure 3a). However, the SOD levels increased in the EPM group (EPM50, 6.37 unit/mg of protein; EPM100, 6.70 unit/mg of protein) compared with the PM_2.5_ group. There were no significant differences between the untreated (Sham) group (7.69 unit/mg of protein), the normal sample (NS) group (7.19 unit/mg of protein), and the NC group.

Reduced glutathione (GSH) levels were reduced in the PM_2.5_ group (87.12%) compared with the NC group (100%) (Figure 3b). However, the reduced GSH levels were reported to increase in the EPM group (EPM50, 93.70%; EPM100, 93.95%) compared with the PM_2.5_ group. There were no significant differences in the Sham group (97.90%), the NS group (99.05%), and the NC group.

Malondialdehyde (MDA) contents were reported to increase in the PM_2.5_ group (1.63 nmole/mg of protein) compared with the NC group (1.13 nmole/mg of protein) (Figure 3c). However, the MDA contents were decreased in the EPM group (EPM50, 1.32 nmole/mg of protein; EPM100, 1.20 nmole/mg of protein) compared with the PM_2.5_ group. There were no significant differences in the Sham group (1.24 nmole/mg of protein), the NS group (1.09 nmole/mg of protein), and the NC group.

To assess the protective effect of EPM on PM_2.5_-induced oxidative stress, the expression levels of proteins related to the Nrf2 signaling pathway (Nrf2, Keap 1, and HO-1) (Figure 3d,e) were measured. As a result, the expression levels of Nrf2 and HO-1 decreased in the PM_2.5_ group (0.83 and 0.82) compared to the NC group (1.00). In contrast, the expression levels of Keap 1 increased in the PM_2.5_ group (2.19) compared with the NC group. In addition, the expression of the Nrf2 signaling pathway was improved in the EPM100 group (1.01, 1.28, and 1.17) compared to the PM_2.5_ group.

### 2.4. EPM Improved Mitochondrial Function in PM_2.5_-Induced Pulmonary Dysfunction

**Figure 4:**
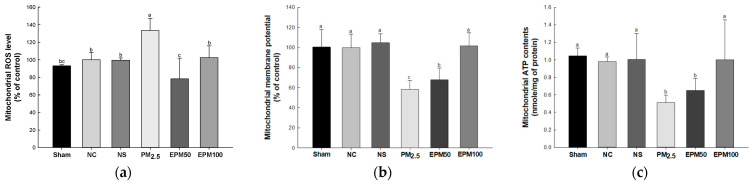
Effect of 40% ethanolic *P. multiflorum* (EPM) extracts on mitochondrial dysfunction in PM_2.5_-induced mice. (**a**) Mitochondrial reactive oxygen species (ROS) levels. (**b**) Mitochondrial membrane potential (MMP) levels. (**c**) Mitochondrial ATP contents. The data were expressed as mean ± SD (*n* = 3). A one-way analysis of variance (ANOVA) test with Duncan’s multiple range tests was conducted. Distinct lowercase letters on the bar graph denote significant differences among groups (*p* < 0.05).

Mitochondrial ROS levels were increased in the PM_2.5_ group (133.35%) compared with the NC group (100%) (Figure 4a). However, the mitochondrial ROS levels decreased in the EPM group (EPM50, 78.40%; EPM100, 102.48%) compared with the PM_2.5_ group. There were no significant differences in the Sham group (93.15%), the NS group (99.51%), and the NC group.

Mitochondrial membrane potential (MMP) was reduced in the PM_2.5_ group (58.12%) compared with the NC group (100%) (Figure 4b). However, MMP was increased in the EPM group (EPM50, 67.99%; EPM100, 101.44%) compared with the PM_2.5_ group. There were no significant differences in the Sham group (100.42%), the NS (104.68%) group, and the NC group.

Mitochondrial ATP contents decreased in the PM_2.5_ group (0.51 nmole/mg of protein) compared with the NC group (0.98 nmole/mg of protein) (Figure 4c). However, the mitochondrial ATP contents increased in the EPM group (EPM50, 0.65 nmole/mg of protein; EPM100, 1.00 nmole/mg of protein) compared with the PM_2.5_ group. There were no significant differences in the Sham group (1.05 nmole/mg of protein), the NS group (1.00 nmole/mg of protein), and the NC group.

### 2.5. EPM Inhibited Inflammation in PM_2.5_-Induced Pulmonary Dysfunction

**Figure 5:**
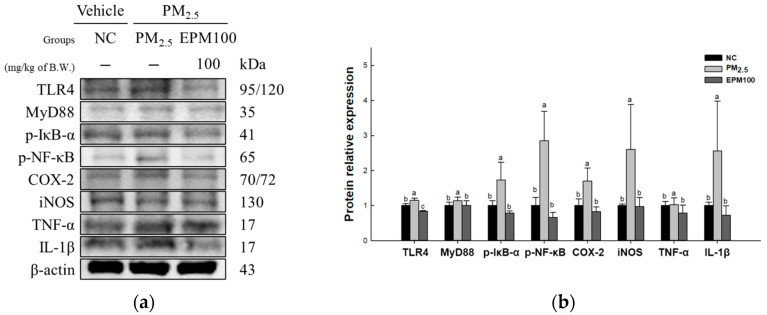
Effect of 40% ethanolic *P. multiflorum* (EPM) extracts on inflammation in PM_2.5_-induced mice. (**a**) Western blot band images. (**b**) Relative expression levels of Toll-like receptor 4 (TLR4), myeloid differentiation primary response 88 (MyD88), phospho-nuclear factor of kappa light polypeptide gene enhancer in B-cells inhibitor-alpha (p-IκB-α), phospho-nuclear factor kappa-light-chain-enhancer of activated B cells (p-NF-κB), cyclooxygenase-2 (COX-2), inducible nitric oxide synthase (iNOS), tumor necrosis factor-α (TNF-α), and interleukin-1 beta (IL-1β) were quantified based on β-actin. The data were expressed as mean ± SD (*n* = 3). A one-way analysis of variance (ANOVA) test with Duncan’s multiple range tests was conducted. Distinct lowercase letters on the bar graph denote significant differences among groups (*p* < 0.05).

To evaluate the protective effect of EPM on PM_2.5_-induced inflammation, the expression levels of proteins related to the TLR4 signaling pathway (TLR4, MyD88, p-IκB-α, p-NF-κB, COX-2, iNOS, TNF-α, and IL-1β) (Figure 5) were measured. As a result, the expression increased in the PM_2.5_ group (1.15, 1.14, 1.73, 1.42, 1.70, 2.60, 1.37, and 4.74) compared to the NC group (1.00). In addition, the expression decreased in the EPM100 group (0.84, 1.01, 0.79, 0.74, 0.82, 0.97, 1.00, and 0.96) compared to the PM_2.5_ group.

### 2.6. EPM Regulates Apoptosis in PM_2.5_-Induced Pulmonary Dysfunction

**Figure 6:**
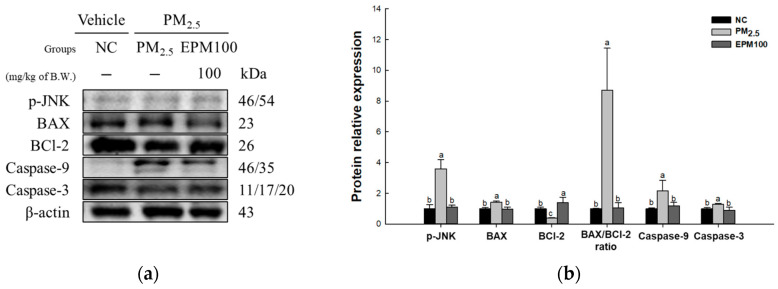
Effect of 40% ethanolic *P. multiflorum* (EPM) extracts on apoptosis in PM_2.5_-induced mice. (**a**) Western blot band images. (**b**) Relative expression levels of phospho-c-Jun N-terminal kinases (p-JNK), BCl-2-associated X (BAX), B-cell lymphoma 2 (BCl-2), BAX/BCl-2 ratio, caspase-9, and caspase-3 were quantified based on β-actin. The data were expressed as mean ± SD (*n* = 3). A one-way analysis of variance (ANOVA) test with Duncan’s multiple range tests was conducted. Distinct lowercase letters on the bar graph denote significant differences among groups (*p* < 0.05).

To assess the protective effect of EPM on PM_2.5_-induced cytotoxicity, the expression levels of proteins related to the JNK signaling pathway (p-JNK, BCl-2, BAX, BAX/BCl-2 ratio, caspase-9, and caspase-3) (Figure 6) were measured. As a result, the expression levels of p-JNK, BAX, BAX/BCl-2 ratio, caspase-9, and caspase-3 increased in the PM_2.5_ group (3.60, 1.42, 8.70, 2.17, and 1.29) compared to the NC group (1.00). In contrast, the expression levels of BCl-2 (0.40) decreased in the PM_2.5_ group compared with the NC group. In addition, the expression of the JNK signaling pathway improved in the EPM100 group (1.11, 1.40, 0.98, 1.06, 1.19, and 0.90) compared to the PM_2.5_ group.

### 2.7. EPM Decreased Fibrosis in PM_2.5_-Induced Pulmonary Dysfunction

**Figure 7:**
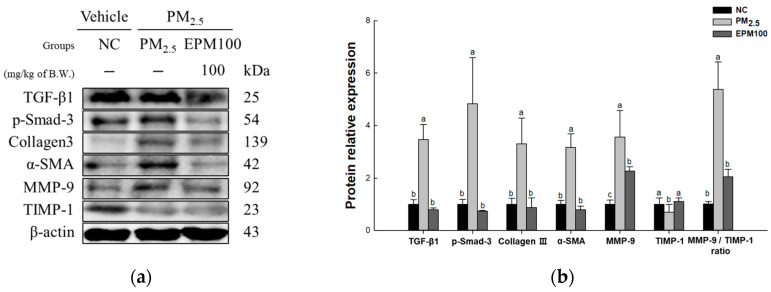
Effect of 40% ethanolic *P. multiflorum* (EPM) extracts on pulmonary fibrosis in PM_2.5_-induced mice. (**a**) Western blot band images. (**b**) Relative expression levels of transforming growth factor beta 1 (TGF-β1), phospho-suppressor of mothers against decapentaplegic-3 (p-Smad-3), Collagen III, alpha-smooth muscle actin (α-SMA), metalloproteinase-9 (MMP-9), tissue inhibitor of matrix metalloproteinase-1 (TIMP-1), and the MMP-9/TIMP-1 ratio were quantified based on β-actin. The data were expressed as mean ± SD (*n* = 3). A one-way analysis of variance (ANOVA) test with Duncan’s multiple range tests was conducted. Distinct lowercase letters on the bar graph denote significant differences among groups (*p* < 0.05).

To investigate the protective effect of EPM on PM_2.5_-induced pulmonary dysfunction, the expression levels of proteins related to the TGF-β1 signaling pathway (TGF-β1, p-Smad-3, Collagen III, α-SMA, MMP-9, TIMP-1, and MMP-9/TIMP-1 ratio) (Figure 7) were measured. As a result, the expression levels of TGF-β1, p-Smad-3, Collagen III, α-SMA, and MMP-9, as well as the MMP-9/TIMP-1 ratio, increased in the PM_2.5_ group (3.47, 4.83, 3.30, 3.17, 3.56, and 5.31) compared to the NC group (1.00). In contrast, the levels of TIMP-1(0.70) expression were found to decrease in the PM_2.5_ group compared with the NC group. In addition, the expression of the TGF-β1 signaling pathway improved in the EPM100 group (0.79, 0.74, 0.87, 0.79, 2.26, 1.11, and 2.02) compared to the PM_2.5_ group.

## 3. Discussion

PM is a major detrimental atmospheric pollutant composed of various biological materials, inorganic elements, and organic compounds, and it is a significant concern due to its high potential to adversely affect respiratory health [1]. In particular, PM_2.5_ enters the bronchial airways through inhalation and induces oxidative stress [3]. Mitochondrial dysfunction and oxidative stress-induced inflammatory responses are implicated in lung tissue injury and the pathogenesis of chronic respiratory conditions such as pulmonary fibrosis and chronic obstructive pulmonary disease (COPD) [9,16]. Accordingly, this research investigated the pulmonary protective properties of EPM in a PM_2.5_-induced murine model of pulmonary dysfunction, as well as exploring its feasibility as a functional food component.

Currently, studies have shown that *P. multiflorum* exhibits protective effects against a range of diseases associated with health conditions, aging, and the regulation of oxidative balance [11,17,18]. Accordingly, UPLC-Q-TOF/MS^E^ analysis was conducted to identify the bioactive compounds present in EPM before initiating cell experiments. As a result of the analysis, EPM was confirmed to contain sucrose, TSG, emodin-1-O-β-glucoside, and emodin-O-(malonyl)-glucopyranoside (Figure 1 and Table 1). These findings align with prior research, indicating that plants belonging to the genus *Polygonum* have been reported to possess a comprehensive selection of anthraquinone constituents such as TSG, rhein, and emodin, along with a variety of phenolic compounds and stilbene [11,17,19,20,21]. In particular, TSG, the major compound of *P. multiflorum*, has been shown to have protective effects on the lungs in an asthma mouse model [19]. Moreover, TSG has been reported to contribute to reducing colitis symptoms by regulating oxidative and nitrosative stress in acute colitis mice [22]. Similarly, EPM treatment improved cytotoxicity and oxidative stress in PM_2.5_-induced A549 and RPMI 2650 cells in our study (Figure 2). These results indicate that anthraquinones, including TSG from *P. multiflorum*, may exhibit various physiological activities. Therefore, this study aimed to explore whether EPM with various bioactive compounds could alleviate the pulmonary dysfunction caused by PM_2.5_ exposure in a mouse model.

ROS generated by PM disrupt metal ion homeostasis, leading to redox imbalance and ROS accumulation, which subsequently contribute to protein modifications, lipid oxidative degradation, and genotoxic damage [23]. Cells exposed to PM_2.5_ demonstrate an increased expression of genes associated with antioxidants that regulate internal balance and counteract oxidative stress [24]. As oxidative stress increases, the degradation regulator Keap1 undergoes direct modulation by cytosolic reactive species, facilitating Nrf2 activation [25]. Activated Nrf2 migrates to the nucleus, where it interacts with ARE/MAF regulatory elements and promotes the transcription of ARE-driven detoxifying enzymes, including HO-1 [26]. HO-1 and its metabolic derivatives exert cytoprotective properties by attenuating the generation of ROS and inhibiting intracellular lipid oxidative degradation [24]. A prior investigation reported that TSG improves SOD and glutathione peroxidase enzymatic functions in both systemic circulation and tissue compartments in a d-galactose-induced murine model of dementia [18]. Another investigation identified that emodin mitigates pulmonary complications linked to acute pancreatic inflammation in mice, with the suppression of inflammasome priming, which is mediated through the Nrf2/HO-1 regulatory axis [27]. In the present investigation, EPM treatment led to elevated SOD activity in the lung tissue, accompanied by a reduction in GSH levels and MDA accumulation (Figure 3a–c). Furthermore, EPM protected against oxidative stress by enhancing the expression of antioxidant-related proteins such as Nrf2 and HO-1, as well as simultaneously inhibiting the expression of Keap1 (Figure 3d,e). The findings suggest that EPM exhibits potential pulmonary-protective properties against PM_2.5_ by regulating antioxidant-related protein expression and reinforcing the antioxidant defense system. This suggests that EPM may be a natural agent that supports the pulmonary antioxidant system by protecting pulmonary function.

Persistent oxidative stress caused by PM_2.5_ interacts with iron–sulfur clusters and ubiquinone in electron transport chain complexes I and III, thereby disrupting electron flow [28]. This disruption inhibits the movement of protons and disrupts the gradient of protons across the inner membrane of the mitochondria, leading to a decrease in MMP and ATP synthesis, thereby affecting cellular energy metabolism [28,29]. The impairment of mitochondrial functions responsible for energy generation, cellular growth, differentiation, and programmed cell death mechanisms leads to the elevated production of ROS, which are a byproduct of respiration that affects the onset and progression of pulmonary diseases [30]. In a previous study regarding the damage to aged cardiomyocytes caused by transient hypoxia, TSG helped restore the protective effect against cardiomyocyte damage by reducing ROS levels and calcium overload, as well as increasing mitochondrial ATP production [31]. Furthermore, TSG protects against mitochondrial dysfunction and apoptosis in SH-SY5Y cells by increasing MMP and ATP levels and decreasing ROS levels and the BAX/BCl-2 ratio [32]. In this study, the administration of EPM protected against mitochondrial dysfunction by downregulating mitochondrial ROS levels and improving MMP levels and ATP content compared with the PM_2.5_ group (Figure 4). These results indicate that EPM mitigates mitochondrial dysfunction by boosting MMP and ATP while reducing ROS. Accordingly, these findings imply that EPM possesses the potential to preserve pulmonary health by mitigating mitochondrial impairment.

Prolonged exposure to PM_2.5_ leads to pulmonary injury, which involves oxidative stress and inflammatory cell infiltration [33]. Redox imbalance and inflammatory cascades are significant factors in developing pulmonary injury, which are caused by PM_2.5_ and the advancement of pulmonary diseases [34]. TLRs are closely related to innate immunity, and the TLR signaling pathway is significant in pulmonary injury and pathogenesis [35]. The activation of TLR4 triggers IκB kinase activation through a MyD88-dependent pathway, which phosphorylates and degrades IκB-α, sequestering NF-κB in the cytoplasm and ultimately allowing the NF-κB complex (p50/p65) to be phosphorylated and transported into the nucleus [36]. Phosphorylated NF-κB subsequently migrates to the cell nucleus, resulting in the upregulation of COX-2, iNOS, TNF-α, and IL-1β [37]. The excessive activation of the TLR4/NF-κB pathway induces the hyperactivation of macrophages, promoting ROS generation due to persistent inflammatory responses, ultimately worsening lung tissue damage [2,6]. Therefore, it is very important to reduce the expression of the inflammatory proteins associated with this pathway. In a previous study, TSG was found to inhibit the p38/NF-κB signaling pathway, which regulates the cytokines involved in immunity, anti-apoptosis, and inflammatory pain, thereby effectively lowering TNF-α expression [38]. In addition, TSG exhibited inflammation-reducing properties through the downregulation of COX-2 and the removal of ROS metabolites [22]. Furthermore, TSG alleviated inflammation in LPS-induced injury by suppressing key cytokines, including IL-1β, COX-2, iNOS, and TNF-α [39]. In this study, the administration of EPM protected the lungs from an inflammatory response by downregulating the TLR4/NF-κB pathway and the expression of inflammatory cytokines (Figure 5). These results suggest that EPM extracts containing TSG can suppress inflammation by inhibiting the expression of inflammation-related proteins. Therefore, EPM can potentially alleviate pulmonary dysfunction by regulating lung inflammation.

Inflammatory cytokines increased due to PM_2.5_ exposure activating transforming growth factor-beta activated kinase 1 (TAK1), which belongs to the mitogen-activated protein kinase (MAP3K) family [34,40]. Activated TAK1 phosphorylates and activates JNK, which is a downstream signaling pathway [40]. Activated JNK stimulates the pro-apoptotic protein BAX, which stimulates cytochrome c release from mitochondria to the cytosol, recruits caspase-9, and activates downstream caspase-3 to induce apoptosis [41]. BCl-2 is an anti-apoptotic protein that plays an essential role in regulating apoptotic signaling [42]. An imbalance of the BAX/BCl-2 ratio stimulates pore formation in the mitochondrial outer membrane, as well as cytochrome complex release [43]. It is important to decrease the expression of apoptosis-related proteins such as JNK and increase the expression of anti-apoptosis-related proteins such as BCl-2. In a previous study using an in vitro ischemic model of oxygen–glucose deprivation followed by reperfusion, TSG inhibited apoptotic signaling pathways by regulating the expression levels of JNK, BCl-2, and Bax [44]. In addition, in the brain tissue of an animal model of chronic inflammatory pain, TSG protected neuronal survival from apoptosis by increasing BCl-2 and decreasing the expression levels of BAX and caspase-3 [38]. In this study, EPM protected cells by inhibiting JNK activation caused by oxidative stress and increasing BCl-2 levels, thereby reducing the expression of apoptosis-related proteins (Figure 6). These results suggest that TSG-rich EPM may help protect the lungs by mitigating PM_2.5_-induced apoptosis through the JNK pathway. Therefore, EPM can potentially be a natural protective agent against pulmonary damage by suppressing the apoptotic pathways triggered by air pollutants.

PM_2.5_ exposure induces inflammatory responses, which trigger apoptosis, ultimately leading to progressive tissue damage and upregulated TGF-β expression [7]. TGF-β serves as a key regulator of fibrosis by stimulating fibroblast differentiation into myofibroblasts and increasing their proliferative capacity, thereby promoting the synthesis, secretion, and deposition of extracellular matrix (ECM) components such as collagen and fibronectin [8]. Additionally, activated TGF-β induces Smad2/3 phosphorylation and facilitates their complex formation with Smad4 [8,45]. Subsequently, the Smad complex moves to the nucleus and functions as a key element that promotes fibrosis-related gene expression, including Collagen III and α-SMA [45]. MMP-9 is a critical enzyme involved in ECM remodeling due to its ability to degrade components such as collagen and elastin [46]. MMP-9 indirectly facilitates fibrosis progression by activating latent TGF-β, thereby enhancing fibroblast differentiation into α-SMA-positive myofibroblasts and promoting ECM deposition [47,48]. The activity of MMP-9 is tightly regulated by the endogenous inhibitor TIMP-1 to maintain tissue homeostasis [46]. TIMP-1 is an endogenous MMP inhibitor that forms an MMP/TIMP complex to prevent MMP activation and maintain tissue homeostasis [49]. When ECM degradation and remodeling are dysregulated during the fibrotic process, tissue damage persists, resulting in the disruption of the MMP-9/TIMP-1 balance [8,50]. This imbalance is particularly aggravated in COPD patients, where MMP-9 overexpression and relative TIMP-1 dysregulation significantly contribute to fibrosis progression [50]. Accordingly, strategies to mitigate fibrosis progression are gaining increasing importance. A prior investigation demonstrated that TSG effectively downregulated TGF-β1 levels and inhibited Smad1/2 [21]. Moreover, an atherosclerotic mouse model exhibited reduced MMP-2 and MMP-9 levels, along with attenuated inflammation, following TSG treatment [51]. In this study, EPM administration suppressed fibrosis-related markers, including TGF-β1, p-Smad3, Collagen III, α-SMA, and MMP-9, while upregulating antifibrotic mediators such as TIMP-1, thereby reducing the MMP-9/TIMP-1 ratio and mitigating lung fibrosis (Figure 7). These results suggest that EPM containing TSG can protect against pulmonary fibrosis triggered by PM_2.5_ by reducing fibrosis-related protein expression, including TGF-β1. Therefore, EPM can potentially be used as a substance that can protect against pulmonary fibrosis induced by oxidative damage and inflammatory responses. However, future studies should be conducted that include a physiological analysis, such as of respiratory function, to better support the biological effects of these findings.

## 4. Materials and Methods

### 4.1. Sample Preparation

Dried *P. multiflorum* was purchased from Seosan, Chungcheongnam-do. *P. multiflorum* was ground and extracted with 40% ethanol at 40 °C for 2 h. The extract was filtered through filter paper (Advantec No. 2 330 mm, Advantec Co., Ltd., Tokyo, Japan). The concentrated extract was obtained through the use of a vacuum rotary evaporator and was subsequently frozen. Subsequently, the frozen product was subjected to a freeze-drying process using a vacuum tray dryer, before being stored at −20 °C.

### 4.2. Physiological Compound Analysis

The EPM was prepared by dissolving it in a 50% methanol solution. Analysis was conducted using UPLC-Q-TOF/MS^E^ (Vion, Waters Corp., Milford, MA, USA) with a BEH C_18_ column (100 mm × 2.1 mm, 1.7 μm; Waters Corp.). The column temperature was maintained at 40 °C, and the flow rate was set at 0.4 mL/min. The mobile phase consisted of 0.1% formic acid in water (A) and 0.1% formic acid in acetonitrile (B), following a linear gradient elution as follows: 1% B (0–1 min), 1–100% B (1–8 min), 100% B (8–9 min), 100–1% B (9–9.5 min), and 1% B (9.5–12 min). The electrospray ionization (ESI) source was used in the negative ion mode. The capillary voltage was set to 3 kV, the cone voltage was set to 40 V, the lamp collision energy was set to 10–30 eV, and the spray pressure was set to 40 psi. Mass spectra were collected over a range of 50–1500 *m*/*z*. Data processing was performed using Waters MassLynx™ software (version 4.1, Waters Corp.).

### 4.3. Cytoprotective Effect of A549 and RPMI2650 Cells

The human lung epithelial cells (A549 cells) used in this study were purchased from the Korean Cell Line Bank (KCLB, Seoul, Republic of Korea), and the human nasal septum epithelial cells (RPMI2650) were purchased from the American Type Culture Collection (Manassas, VA, USA). A549 and RPMI2650 cells were cultured at 5% CO_2_, 37 °C. The A549 cells were cultured in Roswell Park Memorial Institute (RPMI) 1640 medium containing 10% bovine calf serum and 1% penicillin (50 units/mL) and streptomycin (100 μg/mL) (P/S). The RPMI2650 cells were used with minimum essential medium (MEM) Eagle’s medium, which contained 10% fetal bovine serum and 1% P/S.

To evaluate the cell viability, cells were divided into 96-well cell culture plates at 1 × 10^4^ cells/well and were cultured for 24 h. The normal control group and PM_2.5_ group were treated with phosphate-buffered saline (PBS), the positive control group was treated with 200 μM vitamin C, and EPM was treated at different concentrations (2, 5, 10, 20, and 50 μg/mL). After 30 min of treatment, the normal control group was treated with PBS, and all other groups were treated with 100 μM PM_2.5_. PM_2.5_ was purchased as nominal 0–3 micron Arizona test dust (Powder Technology Inc., Arden Hills, MN, USA) and was dissolved in distilled water. After 24 h, 5 mg/mL 3-(4,5-dimethylthiazol-2-yl)-2,5-diphenyltetrazolium bromide (MTT) solution was added to each well for a duration of 2 h. Finally, the formazan was dissolved by treatment with dimethyl sulfoxide, and the absorbance was recorded at 570 nm (measurement) and 655 nm (reference) using a microplate reader (Epoch 2, Bio-Tek Instruments, Inc., Winooski, VT, USA).

To evaluate the inhibitory effect of intracellular ROS, cells were divided into black 96-well cell culture plates at 1 × 10^4^ cells/well and were cultured for 24 h. The normal control group and PM_2.5_ group were treated with PBS, the positive control group was treated with 200 μM vitamin C, and EPM was treated at different concentrations (2, 5, 10, 20, and 50 μg/mL). After 30 min of treatment, the normal control group was treated with PBS, and all other groups were treated with 100 μM PM_2.5_; after 24 h, 2′,7′-dichlorofluorescein diacetate (DCF-DA) solution was added to each well for a duration of 50 min. Finally, the fluorescence intensity was measured at 485 nm (excitation) and 535 nm (emission) using a fluorometer (Infinite F200, Tecan, Männedorf, Switzerland).

### 4.4. Animals and Treatment

BALB/c (6 weeks old, male) mice were obtained from Samtako (Osan, Republic of Korea). The animals were separated into groups of five per cage and were kept under controlled laboratory settings, including a temperature of 22 ± 2 °C, a light/dark cycle of 12 h, and a humidity level of 55%. The animals were randomly assigned to one of six groups—sham (maintained under standard conditions), normal control (NC; control air exposure), normal sample (NS; control air exposure + EPM 100 mg/kg of body weight), PM_2.5_ (PM_2.5_ exposure), EPM50 (PM_2.5_ exposure + EPM 50 mg/kg of body weight), and EPM100 (PM_2.5_ exposure + EPM 100 mg/kg of body weight). Mice were exposed to PM_2.5_ (nominal 0–3 micron Arizona test dust, Arden Hills, MN, USA) at a concentration of 500 µg/m^3^ in a whole-body exposure chamber for 5 h per day, 5 days per week, over a 12-week period. This concentration was set based on the high pollution levels in the real world and previous studies [9,52]. According to the WHO’s global air quality guidelines (AQG), it is recommended to maintain an annual PM_2.5_ exposure below 35 μg/m^3^, and maintain a 24 h exposure below 75 μg/m^3^ [53]. The inorganic components of PM_2.5_ used in this experiment were previously studied by Kim et al. [52]. They included Li, Mg, Al, Cr, Mn, Fe, Cu, Zn, Ba, and Pb Cr at concentrations of 0.02, 6.26, 30.36, 0.01, 0.58, 16.91, 0.05, 0.07, 0.22, and 0.03 mg/g, respectively. The NC and PM_2.5_ groups were orally administered drinking water, and the NS, EPM50, and EPM100 groups were orally administered EPM before chamber exposure. The animal procedures were conducted in accordance with the regulations of the Animal Care and Use Committee of Gyeongsang National University (approval number: GNU-230303-M0039; approval date: 3 March 2023). Following chamber exposure, all mice underwent a 12 h fasting period in preparation for ex vivo testing and were subsequently euthanized using CO_2_ inhalation.

### 4.5. Pulmonary Antioxidant System

To measure SOD levels, an SOD assay kit (Dojindo, Kumamoto, Japan) was used according to the manufacturer’s protocol.

To assess reduced GSH levels, lung tissues were homogenized in 10 mM sodium phosphate buffer containing 1 mM ethylenediaminetetraacetic acid (pH 6.0). The homogenate underwent centrifugation at 10,000× *g* for 15 min, and the supernatant was then combined with 5% metaphosphoric acid, followed by centrifugation at 2000× *g* for 2 min. The collected supernatant was utilized in subsequent analyses. It was then incubated with ο-phthalaldehyde in methanol, 0.65 N sodium hydroxide, and 0.26 M Tris-HCl (pH 7.5) under dark conditions for 15 min. The fluorescence intensity was recorded at 360 nm (excitation) and 430 nm (emission) using a fluorometer (Infinite F200, Tecan).

To investigate MDA contents, the lung tissue was homogenized using PBS, and the obtained homogenate was centrifuged at 2536× *g* for 10 min. The supernatant was combined with 0.67% thiobarbituric acid and 1% phosphoric acid, before being reacted at 95 °C for 1 h. Absorbance was recorded at 532 nm using a spectrophotometer (UV-1800, Shimadzu, Kyoto, Japan).

### 4.6. Pulmonary Mitochondrial Function

To evaluate mitochondrial activity, mouse lung tissue was extracted as described previously [9].

To measure the ROS levels in mitochondria, mitochondrial extracts were reacted with respiration buffer [2 mM KH_2_PO_4_, 20 mM HEPES, 1 mM MgCl_2_, 2.5 mM malate, 5 mM pyruvate, 500 μM ethylene glycol-bis (2-aminoethyl ether)-N,N,N′,N′-tetraacetic acid, 23 mM KCl, and 25 μM DCF-DA]. Then, the reactants were incubated for 20 min (dark room) and measured at 485 nm (excitation) and 535 nm (emission) using a fluorometer (Infinite F200, Tecan).

To evaluate the MMP levels in mitochondria, mitochondrial extracts were reacted with mitochondrial isolation buffer [1 μM JC-1, 5 mM malate, and 5 mM pyruvate] and reacted for 20 min (dark room). Then, the reactants were measured at 530 nm (excitation) and 590 nm (emission) using a fluorometer (Infinite F200, Tecan).

To measure ATP content, a commercial ATP kit (Sigma-Aldrich Chemical Co., Milwaukee, WI, USA) was used according to the manufacturer’s protocol.

### 4.7. Western Blot Analysis

To assess protein expression, lung tissue samples from mice were lysed in a protein extraction buffer (Gene All Biotechnology, Seoul, Republic of Korea) with a 1% protease inhibitor. Proteins were separated using SDS-PAGE and were transferred onto a polyvinylidene difluoride membrane (Millipore, Billerica, MA, USA). The membrane was incubated with the primary antibody at 4 °C for 12 h, and the secondary antibody at room temperature for 1 h. After treatment with ECL solution, luminescence was detected using ChemiDoc (iBright^TM^ CL1000, Invitrogen, Carlsbad, CA, USA). Band intensity was quantified using ImageJ software (version 1.54, NIH, Bethesda, MD, USA) and was normalized to β-actin. Details of the antibodies used are listed in Table 2.

### 4.8. Statistical Analysis

All data were expressed as mean ± standard deviation (SD). Data were statistically analyzed using SAS software (version 9.4, SAS Institute, Cary, NC, USA). The one-way analysis of variance (ANOVA) and Duncan’s multiple range tests were used to assess the statistical significance between multiple groups. Statistical significance was set at *p* < 0.05.

## 5. Conclusions

Overall, our findings demonstrate how EPM alleviates PM_2.5_-induced pulmonary inflammation and fibrosis. Mechanistically, EPM mitigated mitochondrial dysfunction by effectively reducing PM_2.5_-induced ROS generation. In addition, it was associated with the decreased expression of inflammatory, apoptotic, and fibrosis-related factors with the inhibition of the TLR4/TGF-β1 signaling pathway. Our findings suggest that EPM modulates inflammatory responses and fibrosis through the TLR4/TGF-β1 pathway and may be used as a functional food ingredient to protect against PM_2.5_-induced pulmonary injury. Furthermore, these effects of EPM are presumed to result from the complex mixture of bioactive constituents in *P. multiflorum*, including TSG and anthraquinones, although further studies are needed to identify the specific active components.

## Figures and Tables

**Table 1:** Bioactive constituents of 40% ethanolic *P. multiflorum* (EPM) extracts identified using ultra-performance liquid chromatography coupled with quadrupole time-of-flight mass spectrometry (UPLC-Q-TOF/MS^E^).

No.	Retention Time (min)	Parent Ion (*m*/*z*)	MS^E^ Fragment (*m*/*z*)	Compound
1	0.79	341	179, 161, 143, 119	Sucrose
2	4.15	405	243, 225, 215, 149	2,3,5,4′-Tetrahydroxystilbene-2-O-β-D-glucoside
3	4.62	301	283, 259, 268, 151	Unknown
4	4.92	431	269, 240, 225	Emodin-1-O-β-glucoside
5	5.06	517	473, 269, 225	Emodin-O-(malonyl)-glucopyranoside

**Table 2:** Primary and secondary antibody information, as used in this study.

Antibody	Catalog NO.	Manufacturer
β-actin	sc-69879	
TLR4	sc-293072	
MyD88	sc-74532	
p-IκB-α	sc-8404	
p-NF-κB	sc-136548	
TNF-α	sc-33639	
IL-1β	sc-515598	
p-JNK	sc-6254	
BCl-2	sc-7382	Santa Cruz Biotechnology (Dallas, TX, USA)
BAX	sc-7480	
Caspase-9	sc-56076	
Caspase-3	sc-56053	
TGF-β1	sc-130348	
p-Smad-3	sc-517575	
MMP-9	sc-13520	
TIMP-1	sc-21734	
Keap1	sc-514914	
HO-1	sc-136960	
COX-2	ab15191	Abcam (Cambridge, UK)
Collagen III	ab184993	
α-SMA	ab5694	
Nrf2	ab62352	
iNOS	18985-1-AP	Proteintech (Rosemont, IL, USA)
Goat-anti-rabbit IgG	#7074	Cell Signaling Tech (Rosemont, IL, USA)
Goat-anti-mouse IgG	#1724044	Bio-Rad (Richmond, CA, USA)

## Data Availability

The data presented in this study are available on request from the corresponding author.

## Abbreviations

The following abbreviations are used in this manuscript:

p-IκB-α	Phospho-nuclear factor of kappa light polypeptide gene enhancer in B-cells inhibitor-alpha
p-NF-κB	Phospho-nuclear factor kappa-light-chain-enhancer of activated B cells
TNF-α	Tumor necrosis factor-α
IL-1β	Interleukin-1 beta
p-JNK	Phospho-c-Jun N-terminal kinases
BCl-2	B-cell lymphoma 2
BAX	Bcl 2-associated X
p-Smad-3	Phospho-suppressor of mothers against decapentaplegic-3
MMP-9	Metalloproteinase-9
TIMP-1	Tissue inhibitor of matrix metalloproteinase-1
Keap1	Kelch-like ECH-associated protein1
HO-1	Heme oxygenase-1
COX-2	Cyclooxygenase-2
α-SMA	Alpha-smooth muscle actin
Nrf2	Nuclear factor erythroid-2-related factor 2
iNOS	inducible nitric oxide synthase
